# Home- and Car-Based Rules in Foster Care Settings to Reduce Exposure to Secondhand Smoke: Before and after Romanian National Clean Air Legislation

**DOI:** 10.3390/ijerph15081631

**Published:** 2018-08-02

**Authors:** Kristie Foley, Lorand Ferencz, Cristian Meghea, Zoltan Abram, Melinda Pénzes, Andrea Fogarasi-Grenczer, Peter Balazs, Lorand Schmidt

**Affiliations:** 1 Department of Implementation Science, Wake Forest School of Medicine, Medical Center Blvd., Winston-Salem, NC 27157, USA; 2 Department of Hygiene, University of Medicine and Pharmacy Targu Mures, Targu Mures 540139, Romania; lorandferencz@yahoo.com (L.F.); abramzoltan@yahoo.com (Z.A.); 3 Department of Obstetrics, Gynecology and Reproductive Biology Michigan State University, East Lansing, MI 48824, USA; Cristian.Meghea@hc.msu.edu; 4 Institute of Public Health, Semmelweis University, Budapest 1088, Hungary; melindapenzes@gmail.com (M.P.); grenczera@gmail.com (A.F.-G.); balazs-peter@windowslive.com (P.B.); 5 Department of General Directorate of Social Assistance and Child Protection of Mures County, Targu Mures 540139, Romania; schlorand@freemail.hu

**Keywords:** secondhand smoke, low- and middle-income countries, LMIC, vulnerable populations, foster care, orphanage, policy

## Abstract

*Background*: To evaluate changes in smoke free rules in the foster care system after the implementation of the Romanian national clean air law. *Methods*: A repeated cross-sectional, self-administered questionnaire among foster care employees (*n* = 599) was conducted in 58 foster care homes during 2014 (*n* = 295) and 51 homes during 2016 (*n* = 304). We estimated the absolute difference in the proportion of employees who stated that smoke free rules existed before and after national clean air legislation. *Results*: There was an absolute increase in 4 of 5 smoke free measures after the law: bans on non-cigarette tobacco products (*n* = 169 to 206, +10.6%), non-smoking on premises for adults (*n* = 142 to 202, +18.3%), and for children (*n* = 201 to 239, +10.3%), and no smoking in cars to transport children (*n* = 194 to 227, +9%). There was a significant increase in the perception of outdoor bans that prohibit employees from smoking on foster care home premises (AOR 2.24, 95% CI 1.14–4.38). The increase in the perception of indoor smoking bans did not change. *Conclusion*: The national law may have had a spillover influence by strengthening smoke free rules in unregulated spaces. Nonetheless, foster care home rules could be further enhanced, particularly in cars that transport children.

## 1. Introduction

Exposure to secondhand smoke (SHS) has steadily declined in the U.S. due to increasing legislation that disallows smoking indoors [1,2]. Although there has been considerable progress in implementing Article 8 of the Framework Convention on Tobacco Control for the protection of exposure from SHS, 93% of the world’s population still lives in countries with poorly enforced or non-existent clean indoor air laws, most in low- and middle-income countries (LMICs). An estimated 40% of children worldwide are exposed to SHS [3,4,5,6]. 

Opponents of clean air laws argue that national legislation displaces smoking into private spaces, but the research has yet to support this claim [7,8,9]. In a study of European Union countries, tobacco control policy adoption was positively correlated with voluntary home-based smoking bans [7]. A recent review provides further evidence that clean air laws do not displace smoking into the home and have either no impact or a positive impact on home-based smoke free rules [9]. We know little about how national clean air laws affect other voluntary tobacco control rules, such as vehicle bans or non-cigarette tobacco use bans. Romania, the focus of this investigation, ranked 8th of 18 European countries in terms of tobacco control in 2010 with an overall smoking prevalence of 26.5% and 61.5% of households reporting complete in-home smoking bans [10]. 

In 2013–2014, a team of scientists and practitioners from the University of Medicine and Pharmacy, Tirgu-Mures and the child protection authority in Mures County/General Directorate of Social Assistance and Child Protection of Mures County, Romania, launched the first-ever study to understand the prevalence and correlates of smoking behavior and secondhand smoke exposure among children living in foster care in Romania (R01TW009280). The study team reported that almost 1 in 3 foster mothers (31%) and foster fathers (30%) smoked and “parental” smoking was correlated with a 2-to-3 fold increase in cigarette smoking among foster care children [11]. There are little data regarding smoking and secondhand smoke exposure of children living in foster care, in general. In a population-base study of children in Sweden, boys and girls in foster care were 2.96 to 3.84 times more likely to be daily smokers than non-foster care children [12]. In addition, foster care and residential care placement were associated with a greater than 3-fold increase in adult smoking among participants in the British Cohort Study [13]. 

As of 2016, there were more than 57,000 children receiving services through the Romanian National Authority for Child Protection and Adoption of whom 20,156 were living in foster care. The Romanian child protection system began major reform in 2000, transitioning most children from large orphanages to family-like environments (≤12 children). Today, Romania has a hybrid system, with the majority of children living in family care homes, but with some large institutional settings for children who cannot be placed in small family care homes due to space limitations, in order to keep sibling groups intact, and/or due to behavioral problems of the child (personal communication, co-author Lorand Schmidt). While the National Authority prohibited indoor smoking in foster care homes prior to promulgation of its national law, there was no formal mechanism to measure compliance, and there was anecdotal evidence that smoking still occurred in some family care homes. 

In March 2016, Romanian Law no. 349/2002 was amended to reduce SHS exposure by making it illegal to smoke in enclosed public places and outdoor playgrounds. “Enclosed public spaces” must have a roof or ceiling and at least two walls, and includes all health care and educational facilities, government buildings, indoor workplaces, restaurants, public transport stops and vehicles, and indoor and outdoor playgrounds. Our team was instrumental in adding specific language to the legislation that names foster care facilities as “enclosed public spaces” (Article 2, letter m) and prohibits the use of tobacco products inside these facilities (Article 3, paragraph 1). This has important implications for explicitly identifying foster care residents and employees as vulnerable to secondhand smoke exposure. 

The purpose of this research was to assess the changes in smoke free rules in the Romanian foster care system between 2014 and 2016 as the national clean air legislation was implemented to assess whether there was any spillover effect of the law to local smoking bans that would reduce (or increase) exposure to vulnerable children to secondhand smoke.

## 2. Materials and Methods 

Sample: We conducted a repeated cross-sectional, self-administered survey of employees working in foster care settings located in three counties in Romania. Sixty-eight foster care homes participated in the study; 58 participated in the baseline assessment (January 2014–February 2015) prior to passage of the legislation and 51 participated after the legislation was passed (September–December 2016). There were 40 homes that participated in both assessments. Employees were invited to participate if they worked for pay in the foster care home; there were no other inclusion or exclusion criteria. The total number of employee respondents was *n* = 295 and *n* = 304 respectively. The median number of respondents per home was 3 (Min–Max: 1–52, Interquartile Range 2–5). Eighty-two percent of the homes had ≤5 respondents. 

Measures: We collected information on socio-demographic and employment characteristics of respondents, including: age (<30; 30–39; 40–49; 50+), sex (male or female), employee position, and smoking status (current, including daily and non-daily smokers). Additionally, employees were asked if their foster care home had five home-based smoke free rules both before and after the legislation. (1) a rule prohibiting tobacco use indoors; (2–3) a rule prohibiting tobacco use outside on the home’s premises (asked separately for children and employees); (4) a rule prohibiting the use of non-cigarette tobacco products (e.g., e-cigarettes); and (5) a rule prohibiting smoking in vehicles used to transport children. Responses were coded yes, no, I do not know, and missing (<1% of data were missing). Individuals were asked at follow-up if they had participated in a home-based smoking prevention program that was ongoing in Transylvanian foster care homes at the same time (51% said “yes”). 

Analysis: We calculated the difference in proportion of employees who indicated that the home-based smoke free rule existed before and after the clean air legislation. We also conducted multiple logistic regression analyses separately for each of the rules (e.g., prohibiting tobacco indoors, prohibiting tobacco outside on the home’s premise) to determine if there was a statistically significant difference in the proportion of respondents reporting that a smoke free rule existed at baseline versus follow-up. The logistic regressions adjusted for sociodemographic characteristics of the employees, participation in a local smoking prevention program, and clustering in foster care homes in the multivariable model. We computed the pre- and post-legislation effect size using *Cohen’s d*, quantifying the magnitude of difference. Stata 14.0 (StataCorp LLC, College Station, TX, USA) was used for all analyses. 

Human Subjects: This study was approved by the Institutional Review Board (IRB) of the University of Medicine and Pharmacy Targu Mures, Romania (ref. Nr.: 19/29 05.2012). Ethical review board approval was also received from the three County General Directorate Social Assistant and Child Protection in Romania. All employees provided oral consent to participate in the study, and data were recorded without personal identifying information.

## 3. Results

Respondents were disproportionately female (63% baseline and 69% follow-up). The majority were between 40–49 years of age. Most of the respondents were employed as an in-home educator/foster “parent”. Smoking prevalence (30% baseline and 45% follow-up) was higher than the national average for adult current smokers in Romania (25%). 

Prior to and after the legislation, the majority of employees reported that their home had a rule prohibiting indoor smoking (85% and 88%, respectively). There was an absolute increase of 10 to 18 percentage points in the proportion of employees who stated that their foster care prohibits smoking outside on the premises of the foster care home, for children and employees. There was also an absolute increase in the proportion of employees reporting that their home had a ban on non-cigarette tobacco products, from 57.3% to 67.9%. Likewise, there was an absolute increase in the proportion of homes that have a local rule disallowing smoking in vehicles used to transport children—see Table 1.

Effect sizes on promulgating local smoke free rules ranged from 0.11 for indoor smoking bans to 0.50 for employee outdoor smoking bans on the foster care premises. Multiple logistic regressions revealed that there was an independent association of the legislation on whether smoking was allowed for employees and other adults on foster care premises, controlling for participation in the pilot smoking prevention intervention, participant characteristics (gender, age, employee type), and smoking status (Table 1). Significantly more foster care home employees reported that no smoking was allowed on premises after the clean air law was enacted (AOR = 2.23, 95% CI 1.11–4.48). More employees also reported other smoke free rules in foster care homes after the clean air law was enacted (AOR ranging from 1.13 to 1.66), but the correlations were not statistically significant. There was no independent association between the pilot smoking prevention intervention and employee perceptions of smoke free rules, controlling for time, participant characteristics (gender, age, employee type), and smoking status. 

## 4. Discussion

There was an absolute increase in the proportion of employees reporting smoke free rules between 2014 and 2016 in all of the 5 rules measured. This is especially noteworthy, as 4 of 5 of these home-based rules were outside the scope of the national clean air law, with the indoor smoking ban being the only exception. We observed a positive spillover influence of the national clean indoor air law on local, voluntary household smoke free rules.

Nonetheless there remains room for improvement. After the clean air law was enacted, one in four employees still reported that homes allowed smoking in vehicles used to transport foster care children. Fine particulate matter (PM_2.5_) concentrations in cars often exceed WHO air quality standards and concentrations remain high and unhealthy despite opening car windows [14]. Prior research has also shown that exposure to smoking while in the car is associated with an increased risk of smoking initiation among children [15]. Local foster care authorities could consider asking about smoker status as part of the driver screening process and requiring all drivers to abstain from smoking while transporting children. 

While improvements in outdoor bans were noted after the national legislation, one in three and one in five employees reported that smoking was still allowed on foster care home premises for adults and for children, respectively, after the clean air law was enacted. Comprehensive “campus” bans are the recommended strategy to reduce secondhand smoke exposure and reduce tobacco use, but they will be challenging to enforce with an estimated 30% of foster care employees who smoke [11]. A cessation support intervention coupled with such a ban could have the dual benefit of reducing the proportion of smoking employees, while also reducing exposure to SHS among children and non-smoking employees. 

In addition, the increase in the proportion of employees reporting bans on non-cigarette tobacco products is promising. In a recent study, 38.5% of Romanian 9th graders had ever tried e-cigarettes, 31.4% had tried cigars and 21.1% had tried a waterpipe [16]. Similar rates of e-cigarette use have been observed among adolescents in other Romanian and Eastern European locales [17,18]. Recently, Philip Morris announced that its plant in Otopeni (near Bucharest) will begin annual production of “heat-not-burn” tobacco products, serving a very large European and Asian market [19]. By banning non-cigarette tobacco products in foster care at the same time as banning cigarette smoking, children will be discouraged from switching tobacco products to circumvent smoke free rules.

While this study provides evidence that clean air legislation does not deter local smoke free policies that fall outside the scope of national clean air laws, there are several limitations. First, employees may feel obliged to respond affirmatively to the existence of local smoke free rules due to social desirability; we were unable to validate their reports with observational data. Second, we were unable to measure enforcement of the smoke free rules. Third, other unobservable factors (e.g., a national campaign to promote smoke free housing) could have been ongoing at the time of the study, making it impossible to rule out their influence on the changes in adoption of smoke free rules without a control group. Finally, due to ethical guidelines, we could not link the baseline and follow-up surveys to construct a cohort, which would have allowed us to assess changes in individual perceptions of the legislation rather than relying on a repeated cross-sectional analysis. Despite these limitations, this is the first study in an LMIC country to test the relationship of national clean air legislation on different types of home- and car-based smoke free rules within a foster care setting, contributing additional evidence that national clean air laws do not lead to lax smoke free rules in residential settings. 

## 5. Conclusions

Overall, our findings reinforce the scant evidence that national clean air laws do not reduce the likelihood of local home-based bans, and if anything, may encourage homes to adopt additional smoke free rules that go beyond indoor smoking bans. There are several additional strategies that could be considered to further reinforce smoke free rules in Romanian foster care homes, including: training of employees on the existence and importance of adopting comprehensive home- and car-based smoke free rules, establishing a clear strategy for policy communication and enforcement, incentivizing homes that achieve a comprehensive smoke free policy environment, and providing cessation support to employees who want to quit whereby further promoting a smoke free home. All of these strategies are aligned with promoting a healthy environment for children who are especially vulnerable. 

## Figures and Tables

**Table 1:** Employees reporting smoke free rules within Romanian foster care homes before and after the national clean air law.

Home and Car Rules to Reduce Exposure to Secondhand Smoke	Before Clean Air Law: Proportion of Employees in 52 Foster Care Homes Who Agree That the Rule Exists (*n* = 295)	After Clean Air Law: Proportion of Employees in 50 Homes Who Agree That the Rule Exists (*n* = 304)	Absolute Change (Δ)	Effect Size (Cohen’s d)	Adjusted Odds Ratio of Time on the Smoke Free Rule ^a^ (95% CI)
Indoor Smoking Ban	85.4	88.1	+2.7	0.11	1.31 (0.67–2.55)
Ban on non-cigarette tobacco products (e.g., e-cig, hookah)	57.3	67.9	+10.6	0.28	1.45 (0.75–2.82)
No smoking allowed on premises for adults	48.0	66.3	+18.3	0.50	2.23 (1.11–4.48)
No smoking allowed on premises for children	68.2	78.5	+10.3	0.31	1.66 (0.84–3.29)
No smoking in cars used to transport children	65.6	74.6	+9.0	0.29	1.13 (0.69–1.86)

^a^ Analysis adjusted for participation in a smoking prevention program targeting foster care children and participant demographics (gender, age, employee type, smoking status).
